# CD44^+^CD24^−^ prostate cells are early cancer progenitor/stem cells that provide a model for patients with poor prognosis

**DOI:** 10.1038/sj.bjc.6604242

**Published:** 2008-02-12

**Authors:** E M Hurt, B T Kawasaki, G J Klarmann, S B Thomas, W L Farrar

**Affiliations:** 1 Cancer Stem Cell Section, Laboratory of Cancer Prevention, Center for Cancer Research, National Cancer Institute at Frederick (NCI-Frederick), National Institutes of Health, Frederick, MD 21702, USA; 2 Basic Research Program, SAIC-Frederick Inc., National Cancer Institute at Frederick (NCI-Frederick), National Institutes of Health, Frederick, MD 21702, USA

**Keywords:** prostate cancer, tumour stem cells, genomics, CD44, CD24

## Abstract

Recent evidence supports the hypothesis that cancer stem cells are responsible for tumour initiation and formation. Using flow cytometry, we isolated a population of CD44^+^CD24^−^ prostate cells that display stem cell characteristics as well as gene expression patterns that predict overall survival in prostate cancer patients. CD44^+^CD24^−^ cells form colonies in soft agar and form tumours in NOD/SCID mice when as few as 100 cells are injected. Furthermore, CD44^+^CD24^−^ cells express genes known to be important in stem cell maintenance, such as *BMI-1* and *Oct-3/4*. Moreover, we can maintain CD44^+^CD24^−^ prostate stem-like cells as nonadherent spheres in serum-replacement media without substantially shifting gene expression. Addition of serum results in adherence to plastic and shifts gene expression patterns to resemble the differentiated parental cells. Thus, we propose that CD44^+^CD24^−^ prostate cells are stem-like cells responsible for tumour initiation and we provide a genomic definition of these cells and the differentiated cells they give rise to. Furthermore, gene expression patterns of CD44^+^CD24^−^ cells have a genomic signature that is predictive of poor patient prognosis. Therefore, CD44^+^CD24^−^ LNCaP prostate cells offer an attractive model system to both explore the biology important to the maintenance and differentiation of prostate cancer stem cells as well as to develop the therapeutics, as the gene expression pattern in these cells is consistent with poor survival in prostate cancer patients.

Prostate cancer is the third leading cause of cancer-related deaths among men, behind colon and lung cancer and leads all nonskin cancer malignancies. This disease kills an estimated 27 000 American men yearly with an estimated 230 000 new cases diagnosed (American Cancer Society, 2006). Treatment of prostate cancer involves radiotherapy, surgery, hormone therapy, and chemotherapy depending on the stage of the disease (Ngugi, 2007; Ngugi and Magoha, 2007). Individuals who succumb to advanced stages of prostate cancer inevitably become refractory to conventional therapy, resulting in disease progression and death. A growing body of evidence suggests cancer stem cells are the putative mediators of chemotherapy resistance and tumour progression (Dean *et al*, 2005). These resistant cells may be the founder population that causes patient relapse and subsequent metastasis. The cancer stem cell hypothesis suggests the existence of a small subpopulation of cells within the tumour that give rise to differentiated tumour cells. It is thought that the cancer stem cells survive conventional treatment to later re-emerge more resistant to therapy (Reya *et al*, 2001). To date, putative cancer stem cells have been identified in blood (Bonnet and Dick, 1997), brain (Singh *et al*, 2004), breast (Al-Hajj *et al*, 2003), lung (Kim *et al*, 2005), skin (Fang *et al*, 2005), pancreas (Li *et al*, 2007), colon (O'Brien *et al*, 2007; Ricci-Vitiani *et al*, 2007), and prostate (Collins *et al*, 2005; Patrawala *et al*, 2006).

Previous studies also demonstrated that putative breast and brain cancer stem cells displayed a nonadherent phenotype, or ‘mammosphere’ and ‘neurosphere’ quality (Singh *et al*, 2004; Ponti *et al*, 2005, respectively). Mammospheres were enriched with CD44 and low for CD24 surface markers. When cultured in a serum-replacement medium, the spheres maintained their stem markers for months. However, with the addition of serum, the CD44^+^CD24^−^ cells differentiated into cells with an epithelial-like morphology expressing mature markers of myoepithelial and luminal cells (Ponti *et al*, 2005). Neurospheres are also enriched for the extracellular marker, CD133. When putative neuronal stem cells growing in serum-replacement media were exposed to serum *in vitro*, the putative neuronal stem cells differentiated into common medulloblastoma cell types and lost their CD133 expression (Singh *et al*, 2004).

Previously, CD44^+^ prostate cancer cells were shown to have the stem-like properties of increased tumorigenic, clonogenic, and metastatic potential (Patrawala *et al*, 2006). CD44^+^ from immortalised prostate cancer cell lines formed colonies in soft agar and tumours in NOD/SCID mice (Patrawala *et al*, 2006). However, it was concluded in this study that the CD44^+^ prostate cells were a heterogenous population containing both primitive stem cells as well as later progenitor cells. It has also been shown that CD44^+^CD133^+^ integrin *α*2*β*1^high^ cells represent the tumorigenic cells from prostate cancer patients (Collins *et al*, 2005). In breast cancer, previous analysis identified CD44^+^CD24^−^ cells as both clonogenic and tumorigenic (Al-Hajj *et al*, 2003). Recently, work with mouse prostate stem-like cells revealed commonalities between mouse prostate stem-like cells and stem-like cells originating from other tissues, including breast (Lawson *et al*, 2007).

Given this recent evidence, we sought to determine if human prostate CD44^+^CD24^−^ cells defined a population of cancer stem cells. We also sought to determine the global molecular signatures and the differentiation potential of these prostate cancer stem cells, as these were issues not previously studied (Collins *et al*, 2005; Patrawala *et al*, 2006). Here, we identify a rare subpopulation of CD44^+^CD24^−^ prostate cancer cells with stem-like characteristics such as increased clonogenic and tumorigenic properties, and the ability to grow as nonadherent spheres in serum-replacement medium. These ‘prostatospheres’ display stem-like gene signatures with high expression of ‘stemness’ genes including *Oct-3/4*, *BMI-1*, smoothened, and *β-catenin*. Interestingly, addition of serum resulted in the differentiation of the prostatospheres into an adherent monolayer that genotypically resembles the bulk culture of cells. Moreover, tumours from mice injected with CD44^+^CD24^−^ cells phenotypically resembled tumours removed from mice injected with total LNCaP cells, indicating that CD44^+^CD24^−^ cells can give rise to the heterogeneous population present in tumours derived from the LNCaP cell line. Additionally, the CD44^+^CD24^−^ cells contain a molecular signature originally identified in breast tumorigenic cells (Liu *et al*, 2007) that can predict patient outcome not only in breast cancer but also in prostate cancer.

Overall, this study delineates an additional marker for prostate cancer stemness an important step in developing effective therapeutics against these important tumour initiators. Furthermore, we provide a genetic signature of the stem-like cells compared with the differentiated cells to understand the underlying genetic differences of these cells. In addition, the LNCaP CD44^+^CD24^−^ cells are an attractive model system to understand the biology of cancer stem cells and their transition into later progenitor cells. Importantly, the LNCaP CD44^+^CD24^−^ cells provide an *in vitro* model to develop therapeutics because they exhibit a genetic signature reflective of patients that have a poor prognosis.

## MATERIALS AND METHODS

### Cells and media

LNCaP and DU145 cells were obtained from ATCC. LNCaP cells were maintained in RPMI-1640+10% fetal bovine serum (FBS)+2 mM L-glutamine+penicillin and streptomycin, and DU145 cells were maintained in DMEM+10% FBS+2 mM L-glutamine+penicillin and streptomycin. Following cell sorting, cells were maintained in serum-replacement medium consisting of DMEM:F12 plus 10 ng ml^−1^ bFGF, 20 ng ml^−1^ EGF, 5 *μ*g ml^−1^ insulin, and 0.4% BSA. For experiments describing the effects of serum on CD44^+^CD24^−^ cells, 10% FBS was added to the serum-replacement medium for the indicated time.

### Flow cytometric analysis and separation

LNCaP and DU145 cells were detached with trypsin, washed once in FACs buffer (PBS containing 1–2% BSA and 5 mM EDTA), then stained with anti-CD24-FITC (Invitrogen, Carlsbad, CA, USA) and anti-CD44-PE (Invitrogen) using 10 *μ*l of antibody per 10^6^ cells, and incubated at 4°C for 15 min. Following incubation, cells were washed once with FACs buffer. For flow cytometric sorting, cells were resuspended in FACs buffer at 20 × 10^6^ cells ml^−1^ and separated on either an Aria cell sorter (BD Biosciences, San Jose, CA, USA) or a MoFlo High Performance cell sorter (Dako Cytomation, Carpinteria, CA, USA). Live cells were gated on the basis of forward and side scatter, and single cells were gated on the basis of forward scatter and pulse width (Supplementary Figure 1A). Gates were determined by analysis of unstained cells, isotype-specific stains (Supplementary Figure 1B), and single stains (BD Biosciences). The CD44^+^CD24^−^ cells were not assessed for purity due to the low numbers of cells obtained.

### Real-time polymerase chain reaction

The expression of CD133 was measured on CD44^+^CD24^−^ and CD44^+^CD24^−^-depleted cells using Cells-to-C_T_ kit (Applied Biosystems, Foster City, CA, USA) and a StepOne Real-time PCR machine (Applied Biosystems). A relative CT experiment was performed using the TaqMan Gene Expression Assay reagent (Applied Biosystems) for CD133 (PROM1, Hs00195682_m1) and was normalised using GAPDH (Hs99999905_m1) and the supplied enzyme mix in the Cells-to-C_T_ kit.

### Soft agar colony assay

Flow sorted cells were washed once in FACs buffer and 3000 cells were suspended in either serum-replacement medium or DMEM+10% FBS containing 0.4% agarose and overlayed onto a 60-mm dish containing a solidified bottom layer of 0.4% agarose in either serum-replacement or DMEM+10% FBS. Once the top layer solidified, 1 ml of medium was placed on top to keep the plates moist. Plates were incubated for 3 weeks until colonies were visible. The plates were stained with 1 : 75 dilution of 0.33% neutral red solution (Sigma, Saint Louis, MO, USA) at 37°C for 1 h, then counted using an Olympus CK2 microscope (Olympus, Center Valley, PA, USA) and Image Pro Software (MediaCybernetics, Silver Spring, MD, USA).

### Mouse xenograft studies

NCI-Frederick is accredited by AAALAC International and follows the Public Health Service Policy for the Care and Use of Laboratory Animals. Animal Care was provided in accordance with the procedures outlined in the ‘Guide for Care and Use of Laboratory Animals’ (Institute of Laboratory Animal Resources, 1996). Following separation by flow cytometry, cells were placed in serum-replacement medium overnight at a concentration of 1000 cells ml^−1^. Cells were trypsinised, washed once with PBS, then resuspended in serum-replacement medium at 1000 cells per 50 *μ*l, mixed with an equal volume of matrigel (BD Biosciences) and injected subcutaneous into male NOD/SCID mice (Jackson Labs, Bar Harbor, ME, USA). Mice were monitored daily for palpable tumour formation for a total of 159 days. Tumour size was measured with calipers. Tumour volume was determined by the following formula: *V*=*W*^2^ × *L*/2 (Carlsson *et al*, 1983). Once tumours reached greater than 1.5 mm in one dimension, mice were euthanised and tumours were extracted. For flow cytometric analysis, tumours were dissociated with collagenase IV (Sigma) using 200 units ml^−1^ in DMEM:F12 and incubated at 37°C for 1.5 h with mechanical disruption every 30 min. Cells were passed through a 70-*μ*m cell strainer and washed twice with FACs buffer.

### RNA isolation, amplification, and microarray analysis

Total RNA was isolated with Trizol (Invitrogen). Following isolation, RNA was amplified using Ambion's MessageAmp II kit (Ambion, Austin, TX, USA). Twenty-microgram-amplified RNA was labelled in a reverse transcription (RT) reaction with an oligo-dT primer, and either Cy3-dUTP or Cy5-dUTP. Following the RT reaction, the RNA was hydrolysed by incubation with NaOH. Probes were purified with a Microcon YM-30 column (Millipore, Billerica, MA, USA). Oligonucleotide microarrays printed by the NIH Microarray Core Facility were prehybridised in 5 × SSC, 0.1% SDS, and 1% BSA for 1 h at 42°C, washed in distilled water then 100% ethanol, and air dried. Hybridisation of mixed Cy-3- and Cy-5-labelled probes was done in 50% formamide, 10 × SSC, 0.2% SDS overnight at 42°C. After hybridisation, slides were washed in decreasing concentrations of SSC (2 × SSC+0.1% SDS, 1 × SSC, and 0.2 × SSC). Arrays were scanned in a GenePix 4000B scanner and analysed using GenePix Pro (Molecular Devices, Sunnyvale, CA, USA). Data analysis was performed using the centred hierarchical clustering algorithm in Cluster and visualised using TreeView, offered by Michael B Eisen as freeware (http://rana.lbl.gov/EisenSoftware.htm).

## RESULTS

### Identification of a small CD44^+^CD24^−^ subpopulation in prostate cell lines with increased clonogenic properties

**Figure 1:**
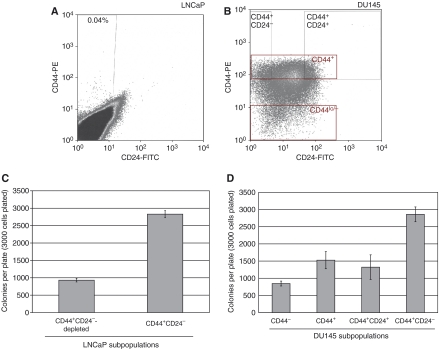
Identification and isolation of clonogenic CD44^+^CD24^−^ cells in prostate cell lines. (**A**) LNCaP cells were analysed by flow cytometry for CD44 and CD24 expression. A small percentage of cells (0.04%) were found to be CD44^+^CD24^−^. (**B**) DU145 cells were analysed by flow cytometry for CD44 and CD24 expression. The gates shown were used for sorting of cells with the indicated phenotypes. The CD44^lo/−^ cells were the lowest 5% of the cells with regards to CD44 staining. (**C**) The soft agar colony-forming efficiencies of both CD44^+^CD24^−^ cells and CD44^+^CD24^−^-depleted LNCaP cells were tested. The experiment was performed in triplicate and repeated, with a representative experiment shown. (**D**) The ability of the CD44^lo/−^, CD44^+^, CD44^+^CD24^+^, and CD44^+^CD24^−^ DU145 subpopulations was assessed for their ability to form colonies in soft agar. A representative experiment of two performed in triplicate is shown.

Given the reported similarity between murine breast and prostate stem cells (Lawson *et al*, 2007) and the recent identification of a CD44^+^CD24^−^ subpopulation of breast cancer cells that have an increased tumorigenic capacity (Al-Hajj *et al*, 2003), we sought to identify and characterise CD44^+^CD24^−^ cells in human prostate cell lines. We identified a CD44^+^CD24^−^ subpopulation with varying abundance in different prostate cancer cell lines. In the LNCaP cell line, the CD44^+^CD24^−^ subpopulation was rare (0.04%) (Figure 1A), whereas the DU145 cell line had a more prevalent CD44^+^CD24^−^ population (7–10%) (Figure 1B). As Collins *et al* (2005) also showed that CD133 is a marker for prostate cancer stem cells, we investigated the CD133 on CD44^+^CD24^−^ and CD44^+^CD24^−^-depleted LNCaP cells. LNCaP CD44^+^CD24^−^ cells have higher expression of CD133 as measured by real-time polymerase chain reaction (Supplementary Figure 1C).

As anchorage-independent growth is an approximation of tumorigenesis and cancer stem cells are thought to be the tumour-initiating cells, we tested the ability of LNCaP CD44^+^CD24^−^ cells and CD44^+^CD24^−^-depleted cells to form colonies in soft agar. There were a similar number of colonies formed by the total LNCaP cells and the CD44^+^CD24^−^-depleted cells (data not shown). Interestingly, the CD44^+^CD24^−^ cells formed approximately three times as many colonies as the CD44^+^CD24^−^-depleted cells (Figure 1C). Thus, nearly all of the 3000 CD44^+^CD24^−^ cells plated were able to initiate a colony (colony-forming efficiency=94%), with an average of 2830 colonies per plate. These results indicate that the CD44^+^CD24^−^ cells represent a near homogeneous population with respect to colony-initiating ability. In addition, the colonies formed faster (visually evident approximately 1 week earlier) and were generally larger in size (approximately 1.5 times) than colonies from CD44^+^CD24^−^-depleted cells (colonies stained at 12 days of culturing are shown in Supplementary Figure 1D). The ability of CD44^+^CD24^−^-depleted cells to form some colonies is likely a result of the incomplete removal of all CD44^+^CD24^−^ cells or may indicate that a portion of these cells still maintain some stem-like properties, although at a lesser extent than the CD44^+^CD24^−^ cells. Using cells purified from the DU145 cell line, we observed a two-fold increase in the ability of CD44^+^CD24^−^ cells in comparison with CD44^+^CD24^+^ (Figure 1D). The fact that CD44^lo/−^ cells also formed colonies at a rate similar to CD44^+^ cells may result from the low level of CD44 expression in the CD44^lo/−^ population, and this population may in fact represent a transient amplifying cell.

### Low numbers of CD44^+^CD24^−^ cells, but not CD44^+^CD24^−^-depleted cells, form tumours in NOD/SCID mice

As the CD44^+^CD24^−^ LNCaP cells formed colonies in soft agar with high efficiency, we compared the ability of the CD44^+^CD24^−^, CD44^+^CD24^−^-depleted cells, and total LNCaP cells to initiate tumours in NOD/SCID mice (Table 1). Either 100 or 1000 cells of each of the populations were mixed with matrigel (1 : 1) and injected subcutaneously into male NOD/SCID mice. As a positive control for tumour formation, both 3 and 5 million total LNCaP cells were also injected. Significantly, injection of 1000 CD44^+^CD24^−^ cells resulted in tumours in 100% (5/5) of mice, whereas 1000 CD44^+^CD24^−^-depleted cells failed to form tumours in any mice (0/5). Injection of 1000 total LNCaP cells initiated a tumour in only one out of five mice. Furthermore, injection of as few as 100 CD44^+^CD24^−^ cells resulted in a single tumour (1/5), whereas 100 CD44^+^CD24^−^-depleted cells or 100 total LNCaP cells were unable to form a tumour (0/5). Tumour formation with DU145 showed an increase in the ability of CD44^+^CD24^−^ cells to form tumours (5/5) in comparison with CD44^+^CD24^+^ cells (3/5). Moreover, while one mouse injected with CD44^+^CD24^+^ cells formed a tumour in a similar time frame as mice injected with CD44^+^CD24^−^ cells, the other two mice that formed tumours had an approximately 1 month longer latency period (Table 1). Also interesting to note is the relative rate of tumour formation of total LNCaP and total DU145. The DU145 cell line, in comparison with the LNCaP cell line, contains a higher percentage of CD44^+^CD24^−^ cells (Figures 1A and B, respectively). In concordance with the higher number of CD44^+^CD24^−^ cells in the DU145 cell line, NOD/SCID mice injected with 3 million DU145 cells formed tumours earlier (approximately 9 days, Table 1) that were 14 times larger at 39 days than NOD/SCID mice injected with LNCaP cells (266.5 *vs* 18.2 mm^3^, respectively). Taken together, this evidence supports the hypothesis that CD44^+^CD24^−^ cells are the tumour-initiating cells in the prostate cancer cell lines. Given that the results obtained with the subpopulations isolated from the LNCaP cell line showed a greater difference in their abilities to both form colonies in soft agar and tumours in NOD/SCID mice, we chose the LNCaP subpopulations for further analyses.

### Tumours removed from mice injected with CD44^+^CD24^−^ cells are differentiated and phenotypically resemble tumours from mice injected with total LNCaP cells

**Figure 2:**
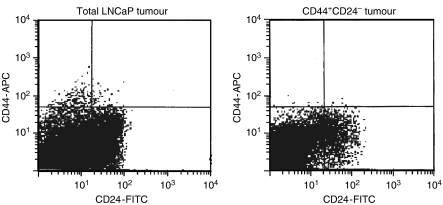
Tumours isolated from mice injected with CD44^+^CD24^−^ cells are phenotypically similar to tumours isolated from mice injected with total LNCaP cells. Tumours were removed from mice injected with either 1000 CD44^+^CD24^−^ cells or 5 × 10^6^ total LNCaP cells. Once dissociated, the cells were stained with anti-CD24-FITC and anti-CD44-PE, and the relative populations of cells were analysed by flow cytometry. The results shown are typical results obtained from the analysis of three tumours from each injection group.

Tumours excised from mice injected with either 1000 CD44^+^CD24^−^ cells or 5 million total LNCaP cells were dissociated, and expression patterns of CD44 and CD24 were analysed by flow cytometry. Figure 2 shows a typical result for each tumour type. Tumours from mice injected with CD44^+^CD24^−^ cells recapitulate the spectrum of cells obtained from tumours from mice injected with total LNCaP cells, including CD44^+^CD24^−^, CD44^−^CD24^+^, and CD44^−^CD24^−^ cells. The total LNCaP tumour showed approximately twice as many CD44^+^CD24^−^ cells compared with the tumour derived from the CD44^+^CD24^−^-purified cells (0.31 and 0.18%, respectively). This may be due to the fact that 5 million total LNCaP cells would be expected to contain approximately 2000 CD44^+^CD24^−^ cells, whereas the tumours removed from the CD44^+^CD24^−^ cells were derived from the injection of only 1000 CD44^+^CD24^−^ cells. The ability of the CD44^+^CD24^−^ cells to give rise to the other cell types contained within the tumours indicates that these are progenitor cells that still harbour the ability to differentiate. Therefore, we have identified a population of LNCaP cells that are stem-like in their biological properties.

### Gene expression analysis of CD44^+^CD24^−^ cells reveals expression of genes that are important for stem cell maintenance

**Figure 3:**
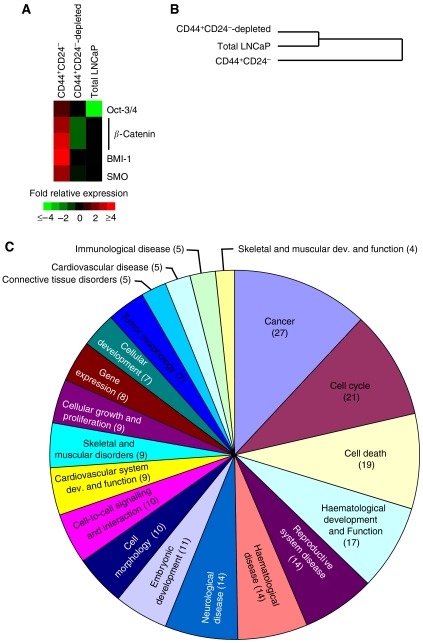
CD44^+^CD24^−^ cells express genes common to other stem-like cells and are genotypically distinct from both total LNCaP and CD44^+^CD24^−^-depleted cells. (**A**) CD44^+^CD24^−^ cells, CD44^+^CD24^−^-depleted cells, and total LNCaP cells were analysed using microarrays for genes expressed by other stem-like cells. CD44^+^CD24^−^ cells show higher expression of the genes analysed. (**B**) The relationship of CD44^+^CD24^−^ cells, CD44^+^CD24^−^-depleted cells, and total LNCaP cells based on gene expression of 5713 genes is shown. While total LNCaP and CD44^+^CD24^−^-depleted cells remain together on a single branch, CD44^+^CD24^−^ cells form a distinct branch, indicating a high degree of difference in their expression patterns for these genes. (**C**) The top 20 functional categories of the genes showing an equal or greater than two-fold change in CD44^+^CD24^−^ cells as compared with both CD44^+^CD24^−^-depleted cells and the total LNCaP cells. The number of genes in each category is indicated in parentheses.

Microarray analysis was performed on CD44^+^CD24^−^, CD44^+^CD24^−^-depleted, and total LNCaP cells. Analysis of genes previously reported to be important for stem cell maintenance show higher expression in CD44^+^CD24^−^ cells relative to the other cell populations (Figure 3A). The transcription factors Oct-3/4 and BMI-1 are important for embryonic stem cell self-renewal (Niwa, 2001) and long-term proliferative capacity of normal haematopoetic and leukaemic stem cells (Valk-Lingbeek *et al*, 2004), and are expressed in cancer stem-like cells (Tai *et al*, 2005; Patrawala *et al*, 2006). An unsupervised analysis, where hierarchical clustering was performed using all genes that were present on 80% of arrays and had a maximum–minimum value of greater than 2 (5713 genes), revealed that CD44^+^CD24^−^ cells have a gene signature that differs significantly from both CD44^+^CD24^−^-depleted and total LNCaP cells (Figure 3B). The large separation of the dendogram likely reflects the significant differences in the biology of the cell types. To determine which genes contribute to the uniqueness of the CD44^+^CD24^−^ cell signature, genes whose expression differs by at least two-fold between the CD44^+^CD24^−^ cells and both the CD44^+^CD24^−^-depleted cells and the total LNCaP cell line were identified. There were 88 genes that were upregulated (Supplementary Table 1) and 255 genes that were downregulated in the CD44^+^CD24^−^ cells (Supplementary Table 2). To determine the relationship of these genes, their functions were analysed using Ingenuity System's Pathways Analysis software. Of the 343 genes showing at least a two-fold change in expression, 156 genes were found and analysed in the Ingenuity database. The top 20 functional categories are shown in Figure 3C, and the complete list of the genes in each category is in Supplementary Table 3. Interestingly, the top categories such as cell-cycle regulation, cell morphology, and cell-to-cell signalling are functions known to differ between stem cells and differentiated cells. It is also interesting to note that one of the categories is embryonic development, suggesting similarities between the biology of CD44^+^CD24^−^ cells and cellular processes important in embryogenesis, including embryonic stem cells.

### CD44^+^CD24^−^ cells form spheres in serum-replacement medium and shift phenotype in the presence of serum

**Figure 4:**
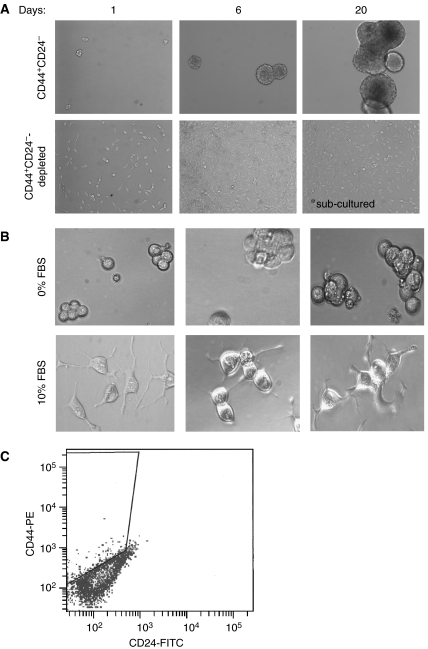
CD44^+^CD24^−^ cells form prostatospheres in serum-replacement media but become adherent and differentiate with the addition of serum. (**A**) CD44^+^CD24^−^ cells and CD44^+^CD24^−^-depleted cells were grown in serum-replacement medium for the indicated time. CD44^+^CD24^−^-depleted cells grew as an adherent monolayer in this medium, reached confluency after 6 days and required subculturing as indicated on the picture at 20 days. However, CD44^+^CD24^−^ cells grew as nonadherent spheres, ‘prostatospheres’ that were not subcultured. Representative fields are shown at × 10 magnification. (**B**) Twenty-four hours after the addition of 10% serum, CD44^+^CD24^−^ cells grew as an adherent monolayer. There are three representative fields shown at × 20 magnification. (**C**) Purified CD44^+^CD24^−^ cells, grown in the presence of serum, shift expression of CD44 and CD24 to look like the original LNCaP culture. CD44^+^CD24^−^ cells were grown in medium containing 10% serum for 7 days and were then analysed for CD44 and CD24 expression by flow cytometry.

Tumour stem cells should have the property of growing as large unattached sphere in the absence of serum (Dontu *et al*, 2003; Lee *et al*, 2006). LNCaP CD44^+^CD24^−^ cells formed ‘prostatospheres’ when placed in serum-replacement medium, whereas CD44^+^CD24^−^-depleted cells grew as an attached monolayer in the same medium (Figure 4A). The prostatospheres proliferate slowly and could be maintained in culture for several weeks without significant alteration of gene expression patterns as compared with the freshly isolated CD44^+^CD24^−^ cells (data not shown). Addition of serum to CD44^+^CD24^−^ prostatospheres induces the cells to grow in an adherent monolayer similar to the CD44^+^CD24^−^-depleted and total LNCaP cells (Figure 4B). Similar adherent morphologies were observed with medium containing 1% (data not shown) or 10% serum. This serum-dependent change in growth pattern was also reported for glioblastoma stem cells (Lee *et al*, 2006). Furthermore, the serum supplemented CD44^+^CD24^−^ cells recapitulated the differentiated phenotype of the total LNCaP cells, with only a small percentage of CD44^+^CD24^−^ cells present, with the majority of the cells being CD44^−^CD24^−^ (Figure 4C). Thus, these data are consistent with the *in vivo* observations of the CD44^+^CD24^−^ cells differentiating into the heterogeneous cells present in the parental LNCaP cell line and demonstrate that CD44^+^CD24^−^ LNCaP stem cells can give rise to differentiated cells that display different surface markers.

### CD44^+^CD24^−^ cells grown in serum genotypically resemble the parental LNCaP cell line

**Figure 5:**
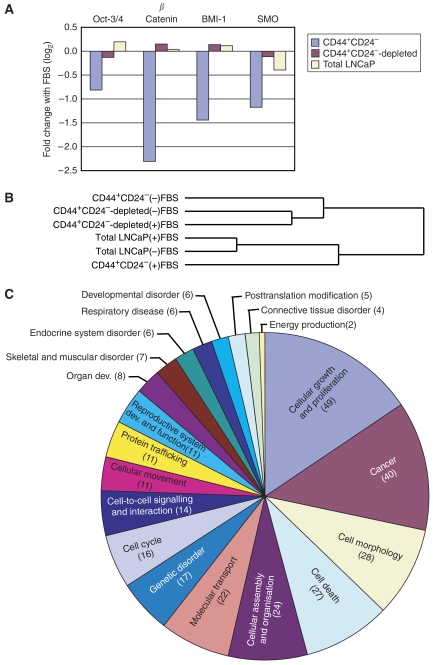
CD44^+^CD24^−^ cells grown in serum-containing medium genomically resemble the parental LNCaP cell line. CD44^+^CD24^−^, CD44^+^CD24^−^-depleted, and total LNCaP cells were grown in either serum-replacement medium alone or serum-replacement medium plus 10% FBS for 1 week. (**A**) Expression of genes important in stem cell biology showed higher expression in freshly isolated CD44^+^CD24^−^ cells. The graph shows the log_2_-fold changes in expression between cells grown in the presence of serum *vs* the same cells grown in the absence of serum. The value provided for *β*-catenin is an average of two array elements. (**B**) Unsupervised hierarchical cluster analysis of arrays was performed. Array tree is based on expression patterns of 4245 genes. (**C**) The top 20 functional categories of genes showing an equal to or greater than two-fold change in CD44^+^CD24^−^ cells grown in serum *vs* without serum as compared with both CD44^+^CD24^−^-depleted cells and the total LNCaP cells. The number of genes in each functional category is indicated in parentheses.

The addition of serum imparts significant morphological and growth properties to the CD44^+^CD24^−^ LNCaP cells. To determine shifts in gene expression underlying the phenotypic changes induced by serum addition, microarrays were performed on CD44^+^CD24^−^, CD44^+^CD24^−^-depleted, and total LNCaP cells grown for 7 days in serum-replacement media with and without 10% FBS added. Figure 3A reveals a serum-induced decrease in expression of stem cell maintenance genes in the CD44^+^CD24^−^ cells; however, the CD44^+^CD24^−^-depleted cells and the total LNCaP cells remained unchanged (Figure 5A). The decreased expression of both *Oct-3/4* and *BMI-1* and the large shift in both phenotype and genotype is consistent with these genes being key regulators in the maintenance of stem cells (Niwa, 2001; Valk-Lingbeek *et al*, 2004). Moreover, unsupervised cluster analysis of 4245 genes show a shift in the overall gene expression of the CD44^+^CD24^−^ cells grown in serum to resemble those of the total LNCaP cell line (Figure 5B). Both of these results are consistent with our previous observations that CD44^+^CD24^−^ cells grown in the presence of serum give rise to the heterogeneous cell types present in the LNCaP cell line. In addition, we determined that 440 genes have at least a two-fold expression change upon addition of serum to the CD44^+^CD24^−^ cells but showed a smaller or no change in both the CD44^+^CD24^−^-depleted and the total LNCaP cells upon addition of serum (Supplementary Table 4). Analysis of cellular functions using Pathway Analysis software, where 189 genes were available in their database, revealed that a large proportion was involved in cellular growth and proliferation (Figure 5C). This is consistent with the increased growth rate that was evident upon serum addition, but is not strictly a serum response as several of these genes change in CD44^+^CD24^−^ cells upon the addition of serum but not in CD44^+^CD24^−^-depleted cells or total LNCaP cells upon the addition of serum. Other cellular function categories showing a high degree of change include cancer, cell morphology, cell death, cellular assembly and organisation, and molecular transport. A complete list of the functions and the genes in each functional category is provided in Supplementary Table 5.

### The invasiveness gene signature is present in the CD44^+^CD24^−^ subpopulation isolated from LNCaP cells

Recently, Liu *et al* (2007) showed that a gene signature termed the invasiveness gene signature (IGS) can predict overall patient survival in breast, medulloblastoma, lung cancer, and prostate cancer. The authors believe that, because the signature originated from breast cancer cells displaying stem-like characteristics, the IGS is predictive of tumours rich in stem cells, and subsequently indicative of a poor prognosis. Since our CD44^+^CD24^−^ cells were isolated from a prostate cancer cell line, we searched for the presence of the IGS in our LNCaP tumour-initiating cells. Of the 187 genes contained in the IGS, 118 of them were represented on the arrays we used to compare CD44^+^CD24^−^ cells to the parental LNCaP cell line. Remarkably, there was a high statistical similarity (Pearson's correlation coefficient=0.59) between our LNCaP CD44^+^CD24^−^ cells compared with the parental cell line and the IGS generated by Liu *et al* from the comparison of breast CD44^+^CD24^−^ cells to normal epithelial cells (Supplementary Table 6). The functions of the genes that show at least an approximate two-fold change in expression in the same direction between the IGS and the CD44^+^CD24^−^ cells *vs* total LNCaP cells (68/118=57%, Pearson's correlation coefficient=0.73) were classified according to function using Pathways Analysis. Of the 68 genes with this correlated expression, 43 were available for analysis of function and are shown in Table 2. These include a large number of transcriptional regulators and kinases as well as numerous genes of unknown function. Thus, there is considerable similarity between breast and prostate tumour-initiating cells, and LNCaP CD44^+^CD24^−^ cells provide a model system that may be useful in understanding the properties of these important tumour-initiating cells, whose underlying biology predicts poor clinical prognosis.

## DISCUSSION

The CD44^+^CD24^−^ prostate cancer cells are highly clonogenic and tumorigenic cells. Moreover, they grow in an anchorage-independent manner and proliferate slowly in serum-replacement medium. Importantly, when these cells are depleted from the LNCaP cell line, the remaining cells are unable to initiate tumour formation in NOD/SCID mice. Furthermore, the tumours that arise in mice injected with purified CD44^+^CD24^−^ LNCaP cells are phenotypically diverse, containing primarily CD44^−^CD24^−^ and CD44^−^CD24^+^ cells and only a small percentage of CD44^+^CD24^−^ cells, similar to tumours derived from injecting the total LNCaP cell line. Likewise, when CD44^+^CD24^−^ cells are grown in serum-containing medium, they phenotypically and genotypically recapitulate the heterogeneity found within the parental cell line. This suggests that the CD44^+^CD24^−^ cells are the clonogenic, tumorigenic cells contained within the LNCaP cell line and that they give rise to the multiple cell types, thereby satisfying the conventional criteria of a cancer stem cell.

Patrawala *et al* (2006) recently showed that CD44^+^ cells derived from prostate cell lines are enriched in tumour progenitor cells, and Collins *et al* (2005) have demonstrated that CD44^+^*α*2*β*1^hi^CD133^+^ cells from patients are highly tumorigenic. Patrawala *et al* (2006) stated that approximately 80% of their CD44^+^ cells were CD44^+^*α*2*β*1^hi^CD133^+^, and they concluded that prostate carcinoma is likely comprised of multiple populations of cells that are able to give rise to tumours with differing efficiencies. Consistent with these findings, the CD44^+^CD24^−^ cells isolated from LNCaP cells also express CD133^+^ (Supplementary Figure 1C). Interestingly, integrin *α*2 expression is increased when the CD44^+^CD24^−^ cells were cultured in serum-containing medium (Supplementary Table 4). The induction of surface integrin *α*2*β*1 remains to be determined due to the low number of cells that are obtained with sorting CD44^+^CD24^−^ cells. While both of the previously identified populations (CD44^+^ and CD44^+^*α*2*β*1^hi^CD133^+^) can give rise to tumours and likely also represent progenitor cells, the increase in the expression of integrin *α*2 in the CD44^+^CD24^−^ cells grown in serum, coupled with the ability of these cells to recapitulate the heterogeneity seen within the LNCaP cell line, suggests that the CD44^+^CD24^−^ cells, which also express CD133, could be a more primitive cell than either the CD44^+^ cells or the CD44^+^*α*2*β*1^hi^CD133^+^ progenitor cells. In agreement with this data, Tang *et al* (2007) have recently discussed a hierarchical organisation of prostate stem cells, which places CD44^+^ cells as an earlier stem cell than the CD44^+^*α*2*β*1^hi^ cells. Our data suggest that the absence of the CD24 marker together with expression of the CD44 marker (i.e., CD44^+^CD24^−^) may identify a very early cancer progenitor cell, or the cancer stem cell itself, but further studies with side-by-side comparisons of these populations are needed to properly assign this hierarchy.

Generally, stem cells are thought to be largely quiescent and to proliferate slowly, eventually giving rise to progenitors that have faster proliferation rates (Tang *et al*, 2007). Thus, the slow proliferation of CD44^+^CD24^−^ cells in culture further supports the idea that this subpopulation of cells most likely represents an early progenitor/stem cell. In addition, one of the largest differences in gene functions between CD44^+^CD24^−^ cells compared with either CD44^+^CD24^−^-depleted or total LNCaP cells was ‘cell cycle’ (Figure 5C), again highlighting the fact that these are largely quiescent cells.

There are several important therapeutic implications in the identification of cancer stem cells. Prostate cancer stem cells are largely undifferentiated and quiescent cells. Meanwhile, conventional chemotherapy generally targets the highly proliferative, more differentiated cells leaving behind the cancer stem cells. This can be highlighted by the fact that breast cancer patients whose tumours have gene expression patterns resembling breast cancer stem cells have a poorer prognosis (Liu *et al*, 2007). Consequently, the identification of the cancer stem cell and an understanding of the mechanisms driving its self-renewal and differentiation pathways are paramount to targeting and to hopefully eradicating the tumour. To develop effective chemotherapeutic agents, we first need to identify the earliest cancer stem cell and understand its biology; tasks that can be difficult with the limited patient material that is often available from prostatectomies. The recent identification of the IGS as a prognostic indicator in prostate cancer and the correlation with its presence in a subset of the LNCaP cell line, suggest that CD44^+^CD24^−^ LNCaP cells can serve as a model system. Thus, the identification of an *in vitro* model system relevant to patient survival provides an invaluable tool, not only to further our understanding of how these cells self-renew and form tumours, but also to develop effective therapeutics.

## Figures and Tables

**Table 1:** Tumour formation of prostate cancer subpopulations in NOD/SCID mice

**NOD/SCID mouse tumour formation with LNCaP subpopulations**		
**LNCaP subpopulation**	**No. of mice with palpable tumours**	**Average time to tumour formation (days)**
1000 CD44^+^CD24^−^	5/5	81 (±12)
100 CD44^+^CD24^−^	1/5	159
1000 CD44^+^CD24^−^-depleted	0/5	—
100 CD44^+^CD24^−^-depleted	0/5	—
1000 total LNCaP	1/5	89
100 total LNCaP	0/5	—
5 × 10^6^ total LNCaP	3/3	30.66 (±4)
^*^3 × 10^6^ total LNCaP	4/4	27.5 (±4)
		
**NOD/SCID mouse tumour formation with DU145 subpopulations**		
**DU145 subpopulation**	**No. of mice with palpable tumours**	**Average time to tumour formation (days)**
100 CD44^+^	5/5	80 (±13)
100 CD44^−^	2/5	86 (±18)
100 CD44^+^CD24^−^	5/5	80 (±3)
100 CD44^+^CD24^+^	3/5	82 (+39)
^*^3 × 10^6^ total DU145	4/4	18 (±5)

Male NOD/SCID mice were injected subcutaneously with the indicated cell phenotype and monitored for tumour formation. The table lists the subpopulations tested, the total number of mice with palpable tumours, and the average number of days until palpable tumour formation. A control of 5 million LNCaP cells was used. To compare the relative tumour-forming abilities of LNCaP and DU145, 3 million cells of each cell line was tested in an experiment independent of the experiment in which the tumour-forming ability of the various subpopulations was tested (designated by the asterisk).

**Table 2:** Classification of genes showing similar regulation between the IGS and the comparison of CD44^+^CD24^−^ cells and total LNCaP cells

**Family**	**Genes**
Cytokine	*CXCL2, IL3, IL4, PBEF1, SCYE1, TNF*
Enzyme	*ARIH1, ATIC, CHPT1, CPT1A, DNMT3A, DNMT3B, ELP3, ERCC5, GAPDH, ICMT, LDHA, LDHB, PRIM2A, RALBP1, UBE2D2, WWOX*
G-protein-coupled receptor	*ADORA2B, OPRK1*
Growth factor	*FIGF, NRG1, TDGF1, TGFB1*
Ion channel	*CFTR, CLCN3*
Kinase	*CSNK1D, CSNK2A1, ERBB4, ERN1, ETNK1, NUAK2, PGK1, PIM2, WEE1*
Peptidase	*ADAM10, ADAM19, CASP1, CASP4, CASP8, F7*
Phosphatase	*NUDT5, PPP1R12C*
Transcription regulator	*BRF1, CDKN2C, CEBPG, CTNNB1, DNMT3L, E2F4, ELF4, ELL2, ETS1, FOS, ISGF3G, KLF10, MYC, NFYB, NKX3-1, RELA, SIM1, XBP1*
Translation regulator	*EIF4E2*
Transmembrane receptor	*MSR1, SFRP1, TNFRSF10B*
Transporter	*APOC2, BCAP31, GOPC, NUP37, NUP43, NUP107, NUP133, PLP2, SEC13L1, STX6*
Others	*AIM1, ARPC2, ARPC4, ARPC5, C4ORF7, C6ORF107, CDC2B, CDKN1B, CNOT4, COL4A2, CSTF1, ELP4, ELP5, ELP6, EMP1, FRAT1, GABARAPL1, HSPA2, HSPE1, IER5, IKBKAP, ISG15, JTV1, ITGB6, KLHL20, MAPT, MLF1, MT1A, NUP85, PLAA, RAD23B, RAD23A, S100A6, SCGN, SERPINB5, SERPINE2, SFTPA1, SNRPN, SWAP70, STATIP1, TAC1, THY1, TOB2, TPD52, VIL2*

IGS=invasiveness gene signature.

IGS genes that showed a 1.8-fold or greater regulation in the comparison of CD44^+^CD24^−^ cells *vs* total LNCaP cells were identified. Of the 68 identified, 42 of them were available in Ingenuity Pathways Analysis databases for analysis of gene function. The functions and the genes identified for those functions are listed.

## References

[bib1] Al-Hajj M, Wicha MS, Benito-Hernandez A, Morrison SJ, Clarke MF (2003) Prospective identification of tumorigenic breast cancer cells. Proc Natl Acad Sci USA 100: 3983–3988 12629218 10.1073/pnas.0530291100 PMC153034

[bib2] American Cancer Institute (2006) Prostrate Cancer Facts and Figures 2006. Ohio: American Cancer Society Inc

[bib3] Bonnet D, Dick JE (1997) Human acute myeloid leukemia is organized as a hierarchy that originates from a primitive hematopoietic cell. Nat Med 3: 730–737 9212098 10.1038/nm0797-730

[bib4] Carlsson G, Gullberg B, Hafstrom L (1983) Estimation of liver tumor volume using different formulas – an experimental study in rats. J Cancer Res Clin Oncol 105: 20–23 6833336 10.1007/BF00391826 PMC12252809

[bib5] Collins AT, Berry PA, Hyde C, Stower MJ, Maitland NJ (2005) Prospective identification of tumorigenic prostate cancer stem cells. Cancer Res 65: 10946–10951 16322242 10.1158/0008-5472.CAN-05-2018

[bib6] Dean M, Fojo T, Bates S (2005) Tumour stem cells and drug resistance. Nat Rev Cancer 5: 275–284 15803154 10.1038/nrc1590

[bib7] Dontu G, Abdallah WM, Foley JM, Jackson KW, Clarke MF, Kawamura MJ, Wicha MS (2003) *In vitro* propagation and transcriptional profiling of human mammary stem/progenitor cells. Genes Dev 17: 1253–1270 12756227 10.1101/gad.1061803 PMC196056

[bib8] Fang D, Nguyen TK, Leishear K, Finko R, Kulp AN, Hotz S, Van Belle PA, Xu X, Elder DE, Herlyn M (2005) A tumorigenic subpopulation with stem cell properties in melanomas. Cancer Res 65: 9328–9337 16230395 10.1158/0008-5472.CAN-05-1343

[bib9] Institute of Laboratory Animal Resources CoLSNRC (1996) Guide for Care and Use of Laboratory Animals. Washington, DC: National Academy Press

[bib10] Kim CF, Jackson EL, Woolfenden AE, Lawrence S, Babar I, Vogel S, Crowley D, Bronson RT, Jacks T (2005) Identification of bronchioalveolar stem cells in normal lung and lung cancer. Cell 121: 823–835 15960971 10.1016/j.cell.2005.03.032

[bib11] Lawson DA, Xin L, Lukacs RU, Cheng D, Witte ON (2007) Isolation and functional characterization of murine prostate stem cells. Proc Natl Acad Sci USA 104: 181–186 17185413 10.1073/pnas.0609684104 PMC1716155

[bib12] Lee J, Kotliarova S, Kotliarov Y, Li A, Su Q, Donin NM, Pastorino S, Purow BW, Christopher N, Zhang W, Park JK, Fine HA (2006) Tumor stem cells derived from glioblastomas cultured in bFGF and EGF more closely mirror the phenotype and genotype of primary tumors than do serum-cultured cell lines. Cancer Cell 9: 391–403 16697959 10.1016/j.ccr.2006.03.030

[bib13] Li C, Heidt DG, Dalerba P, Burant CF, Zhang L, Adsay V, Wicha M, Clarke MF, Simeone DM (2007) Identification of pancreatic cancer stem cells. Cancer Res 67: 1030–1037 17283135 10.1158/0008-5472.CAN-06-2030

[bib14] Liu R, Wang X, Chen GY, Dalerba P, Gurney A, Hoey T, Sherlock G, Lewicki J, Shedden K, Clarke MF (2007) The prognostic role of a gene signature from tumorigenic breast-cancer cells. N Engl J Med 356: 217–226 17229949 10.1056/NEJMoa063994

[bib15] Ngugi PM (2007) An update on the treatment of advanced prostate cancer. East Afr Med J 84: S36–S39 18154201 10.4314/eamj.v84i9.9560

[bib16] Ngugi PM, Magoha GA (2007) The management of early prostate cancer: a review. East Afr Med J 84: S24–S30 18154199 10.4314/eamj.v84i9.9558

[bib17] Niwa H (2001) Molecular mechanism to maintain stem cell renewal of ES cells. Cell Struct Funct 26: 137–148 11565806 10.1247/csf.26.137

[bib18] O'Brien CA, Pollett A, Gallinger S, Dick JE (2007) A human colon cancer cell capable of initiating tumour growth in immunodeficient mice. Nature 445: 106–110 17122772 10.1038/nature05372

[bib19] Patrawala L, Calhoun T, Schneider-Broussard R, Li H, Bhatia B, Tang S, Reilly JG, Chandra D, Zhou J, Claypool K, Coghlan L, Tang DG (2006) Highly purified CD44^+^ prostate cancer cells from xenograft human tumors are enriched in tumorigenic and metastatic progenitor cells. Oncogene 25: 1696–1708 16449977 10.1038/sj.onc.1209327

[bib20] Ponti D, Costa A, Zaffaroni N, Pratesi G, Petrangolini G, Coradini D, Pilotti S, Pierotti MA, Diadone MG (2005) Isolation and *in vitro* propagation of tumorigenic breast cancer cells with stem/progenitor cell properties. Cancer Res 65: 5506–5511 15994920 10.1158/0008-5472.CAN-05-0626

[bib21] Reya T, Morrison SJ, Clarke MF, Weissman IL (2001) Stem cells, cancer, and cancer stem cells. Nature 414: 105–111 11689955 10.1038/35102167

[bib22] Ricci-Vitiani L, Lombardi DG, Pilozzi E, Biffoni M, Todaro M, Peschle C, De MR (2007) Identification and expansion of human colon-cancer-initiating cells. Nature 445: 111–115 17122771 10.1038/nature05384

[bib23] Singh SK, Hawkins C, Clarke ID, Squire JA, Bayani J, Hide T, Henkelman RM, Cusimano MD, Dirks PB (2004) Identification of human brain tumour initiating cells. Nature 432: 396–401 15549107 10.1038/nature03128

[bib24] Tai MH, Chang CC, Kiupel M, Webster JD, Olson LK, Trosko JE (2005) Oct4 expression in adult human stem cells: evidence in support of the stem cell theory of carcinogenesis. Carcinogenesis 26: 495–502 15513931 10.1093/carcin/bgh321

[bib25] Tang DG, Patrawala L, Calhoun T, Bhatia B, Choy G, Schneider-Broussard R, Jeter C (2007) Prostate cancer stem/progenitor cells: identification, characterization, and implications. Mol Carcinog 46: 1–14 16921491 10.1002/mc.20255

[bib26] Valk-Lingbeek ME, Bruggeman SW, van Lohuizen M (2004) Stem cells and cancer; the polycomb connection. Cell 118: 409–418 15315754 10.1016/j.cell.2004.08.005

