# Supplementary figures and images for: CD44+CD24− prostate cells are early cancer progenitor/stem cells that provide a model for patients with poor prognosis
Source: Br J Cancer. 2008 Feb 12;98(4):756–65. doi: 10.1038/sj.bjc.6604242 (PMC2259168; doi:10.1038/sj.bjc.6604242)

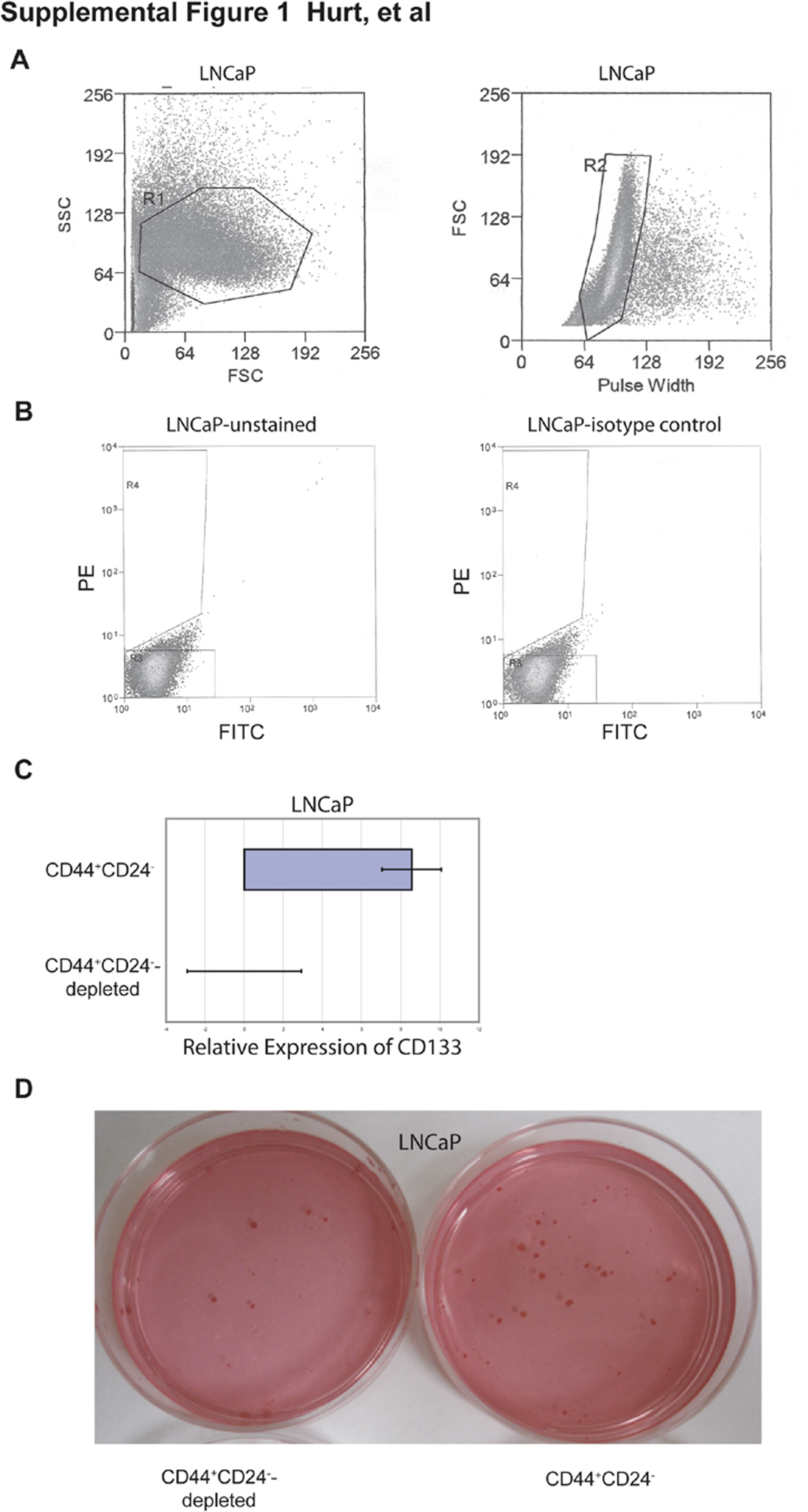

Supplement: Supplementary Figure 1 [file 6604242x1.tif]
